# Supplementary material for: Cecytb-2, a Cytochrome b561 Homolog, Functions as an Ascorbate-Specific Transmembrane Ferric Reductase at Intestinal Lumens of Caenorhabditis elegans
Source: Biomolecules. 2025 Sep 29;15(10):1385. doi: 10.3390/biom15101385 (PMC12563681; doi:10.3390/biom15101385)
Supplement: Supplementary file 1 [file biomolecules-15-01385-s001.zip › biomolecules-3863761-supplementary.pdf]

Supplementary Materials for

Cecytb-2, a Cytochrome b_{561} Homolog, Functions as an Ascorbate-Specific Transmembrane Ferric Reductase at Intestinal Lumens of *Caenorhabditis elegans*

Masahiro Miura, Misaki Fukuzawa, Hiroshi Hori, Kazuo Kobayashi, Mariam C. Recuenco and Motonari Tsubaki*

(a) Cecytb-2 （*F39G3.5*） 251 a.a. 28,127 Da (av.)

MSSDSRLGNARSFNRFDIILSITHFVGFITICLNGYFLNTFKNGLAWPSKVKNGDNKALGKRGDQL
HAFLMILAFIYFQGEALLAYRLYRYDAKIISKLL**H**TALHIIAIGLGITALTVIIMSTNNAGWNNFT
SV**H**SWIGICLLSVYLVQFSFGFLTYLCPCSPGKYRARLMPI**H**RAVGVGCFIVACVQCCLGYGNILL
EDQPGCFSDLSCKNRIEYVGAFSVMFIILYTLLVLALIIPVPWRREKTPDELK

(b) Cecytb-2-H_6（recombinant protein） 259 a.a. 29,108 Da (av.)

MSSDSRLGNARSFNRFDIILSITHFVGFITICLNGYFLNTFKNGLAWPSKVKNGDNKALGKRGDQL
HAFLMILAFIYFQGEALLAYRLYRYDAKIISKLL**H**TALHIIAIGLGITALTVIIMSTNNAGWNNFT
SV**H**SWIGICLLSVYLVQFSFGFLTYLCPCSPGKYRARLMPI**H**RAVGVGCFIVACVQCCLGYGNILL
EDQPGCFSDLSCKNRIEYVGAFSVMFIILYTLLVLALIIPVPWRREKTPDELKSAHHHHHH

(c) DHFR-C2Ct 220 a.a. 25,293 Da (av.)

MRGSHHHHHHGSGIMVRPLNSIVAVSQNMGIGKNGDLPWPPLRNEFKYFQRMTTTSSVEGKQNLVI
MGRKTWFSIPEKNRPLKDRINIVLSRELKEPPRGAHFLAKSLDDALRLIEQPELASKVDMVWIVGG
SSVYQEAMNQPGHLRLFVTRIMQEFESDTFFPEIDLGKYKLLPEYPGVLSEVQEEKGIKYKFEVYE
KKGSDLGSPVPWRREKTPDELK

Figure S1. Amino acid sequences of Cecytb-2 proteins used in the present study

For the native Cecytb-2 protein **(a)**, the putative heme ligands (four His residues; His67, His101, His135, and His174) are indicated in bald face with underlined. For the recombinant Cecytb-2-H_6 protein **(b)**, a linker sequence (Ser-Ala) followed by a 6xHis-tag sequence (i.e., SAHHHHHH) (underlined) was fused at the C-terminus of the native Cecytb-2 protein.

In DHFR-C2Ct protein **(c)**, the Cecytb-2 C-terminus peptide (PVPWRREKTPDELK) (underlined) was fused with dihydrofolate reductase (DHFR) on its C-terminus whereas a 6xHis-tag sequence was fused on its N-terminus.

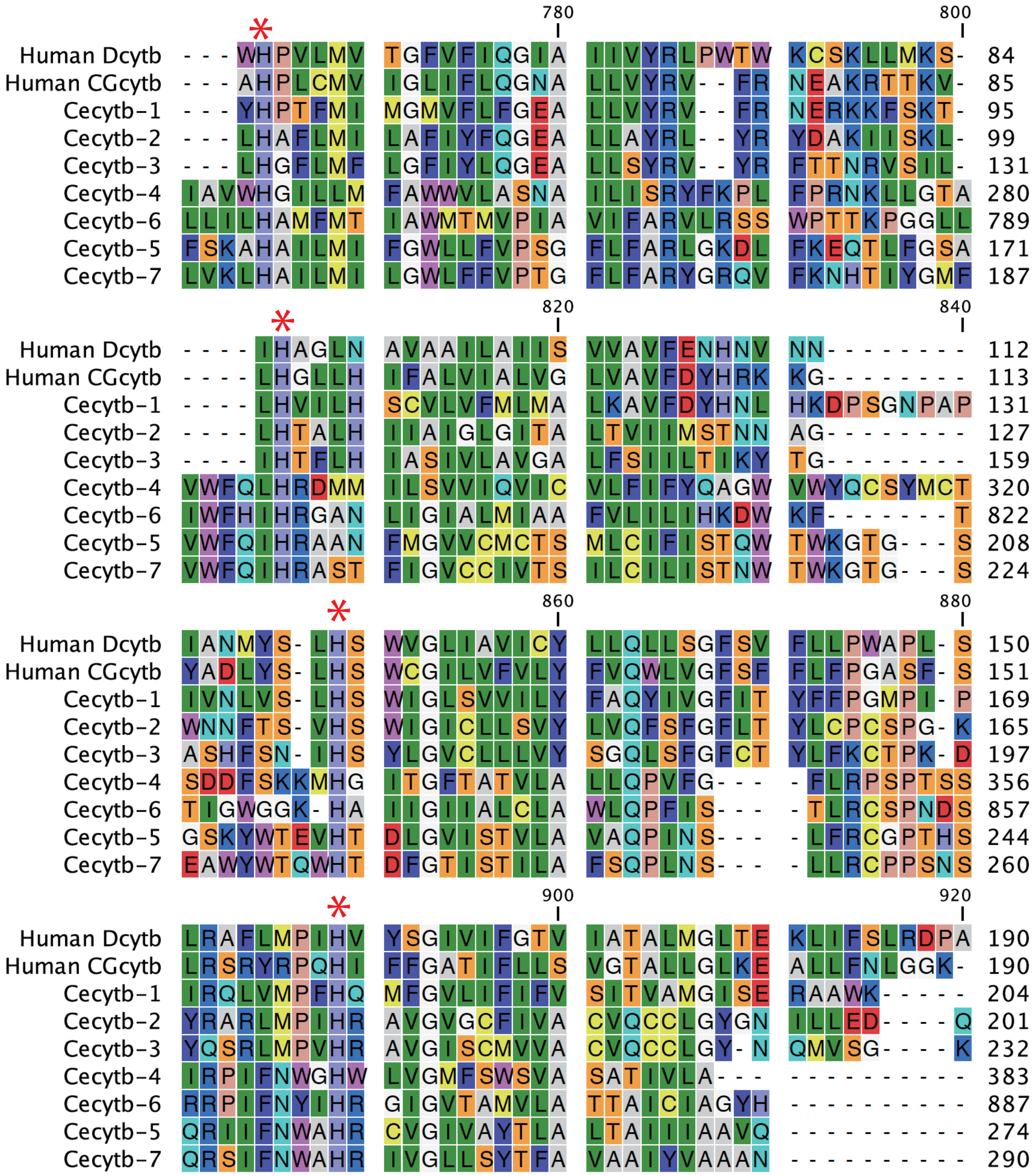

780
800
Human Dcytb --- WHPVLMV TGFVFIQGIA IIVYRLPWTW KCSKLLMKS- 84
Human CGcytb --- AHPLCMV IGLIFLQGNA LLVYRV--FR NEAKRTTKV- 85
Cecytb-1 --- YHPTFMI MGMVFLFGEA LLVYRV--FR NERKKFSKT- 95
Cecytb-2 --- LHAFLMI LAFIYFQGEA LLAYRL--YR YDAKIISKL- 99
Cecytb-3 --- LHGFLMF LGFIYLQGEA LLSYRV--YR FTTNRVSIL- 131
Cecytb-4 IAVWHGILLM FAWWVLASNA ILISRYFKPL FPRNKLLGTA 280
Cecytb-6 LLILHAMFMT IAWMTMVPIA VIFARVLRSS WPTTKPGGLL 789
Cecytb-5 FSKAHAILMI FGWLLFVPSG FLFARLGKDL FKEQTLFGSA 171
Cecytb-7 LVKLHAILMI LGWLFFVPTG FLFARYGRQV FKNHTIYGMF 187
820
840
Human Dcytb ----IHAGLN AVAAILAIIS VVAVFENHNV NN-------- 112
Human CGcytb ----LHGLLH IFALVIALVG LVAVFDYHRK KG-------- 113
Cecytb-1 ----LHVILH SCVLVFMLMA LKAVFDYHNL HKDPSGNPAP 131
Cecytb-2 ----LHTALH IIAIGLGITA LTVIIMSTNN AG-------- 127
Cecytb-3 ----IHTFLH IASIVLAVGA LFSIILTIKY TG-------- 159
Cecytb-4 VWFQLHRDMM ILSVVIQVIC VLFIFYQAGW VWYQCSYMCT 320
Cecytb-6 IWFHIHRGAN LIGIALMIAA FVLILIHKDW KF-------T 822
Cecytb-5 VWFQIHRAAN FMGVVCMCTS MLCIFISTQW TWKGTG---S 208
Cecytb-7 VWFQIHRAST FIGVCCIVTS ILCILISTNW TWKGTG---S 224
860
880
Human Dcytb IANMYS-LHS WVGLIAVICY LLQLLSGFSV FLLPWAPL-S 150
Human CGcytb YADLYS-LHS WCGILVFVLY FVQWLVGFSF FLFPGASF-S 151
Cecytb-1 IVNLVS-LHS WIGLSVVILY FAQYIVGFIT YFFPGMPI-P 169
Cecytb-2 WNNFTS-VHS WIGICLLSVY LVQFSFGFLT YLCPCSPG-K 165
Cecytb-3 ASHFSN-IHS YLGVCLLLVY SGQLSFGFCT YLFKCTPK-D 197
Cecytb-4 SDDFSKKMHG ITGFTATVLA LLQPVFG--- -FLRPSPTSS 356
Cecytb-6 TIGWGGK-HA IIGIIALCLA WLQPFIS--- -TLRCSPNDS 857
Cecytb-5 GSKYWTEVHT DLGVISTVLA VAQPINS--- -LFRCGPTHS 244
Cecytb-7 EAWYWTQWHT DFGTISTILA FSQPLNS--- -LLRCPPSNS 260
900
920
Human Dcytb LRAFLMPIHV YSGIVIFGTV IATALMGLTE KLIFSLRDPA 190
Human CGcytb LRSRYRPQHI FFGATIFLLS VGTALLGLKE ALLFNLGGK- 190
Cecytb-1 IRQLVMPFHQ MFGVLIFIFV SITVAMGISE RAAWK----- 204
Cecytb-2 YRARLMPIHR AVGVGCFIVA CVQCCLGYGN ILLED----Q 201
Cecytb-3 YQSRLMPVHR AVGISCMVVA CVQCCLGY-N QMVSG----K 232
Cecytb-4 IRPIFNWGHW LVGMFSWSVA SATIVLA--- ---------- 383
Cecytb-6 RRPIFNYIHR GIGVTAMVLA TTAICIAGYH ---------- 887
Cecytb-5 QRIIFNWAHR CVGIVAYTLA LTAIIIAAVQ ---------- 274
Cecytb-7 QRSIFNWAHR IVGLLSYTFA VAAIYVAAAN ---------- 290

Figure S2. Multiple alignments of amino acid sequences of human duodenal cytochrome b_{561} (Dytb), human adrenal cyotochrome b_{561} (hCGcytb) and seven homologs in *C. elegans*.

Multiple alignments of human duodenal cytochrome b_{561} (Dcyb), human adrenal cytochrome b_{561} (CGcytb) and their seven homologs in *C. elegans*, including Cecytb-2, were conducted using CLC-Main Workbench software (v. 6.8) (CLC Bio) and only their central parts were shown. In our previous report, we suggested that there are total of six b561 family members in *C. elegans* and one of them, b561C.ele5, contains two b561 domains (C.ele5-1 and C.ele5-2) (Tsubaki et al., 2005)[5]. However, later studies indicated that each of the b561 domains of b561C.ele5 is rather expressed as a distinct protein (gene names; *M03A1.3* and *M03A1.8*). We have confirmed the expression of the former gene as a holo-form of the b561 family protein, Cecyty-5 (Hirano et al., unpublished observation). For the latter gene, we named its gene product as Cecyt-7. Thus, there are total of seven b561 family members in *C. elegans*.

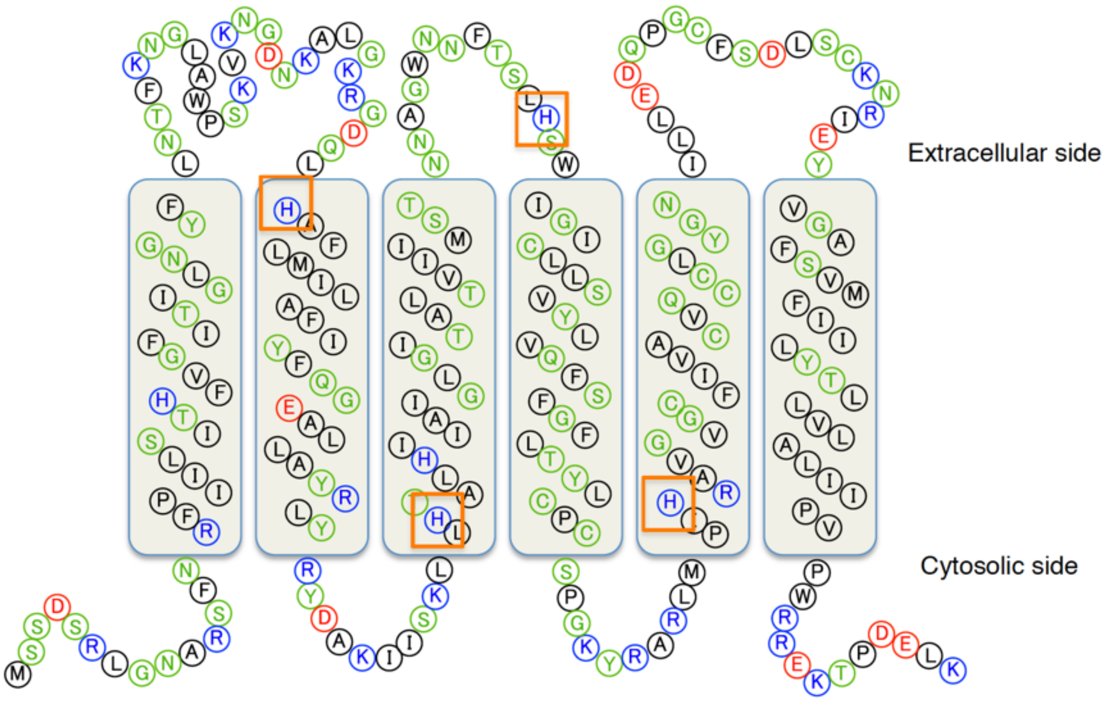

Figure S3. A schematic transmembrane structural model of Cecytb-2 protein.

Hydropathy plot analysis of Cecytb-2 protein indicated that this protein is likely to have six transmembrane α-helices and two pairs of fully-conserved His residues (His67, His101, His135, and His174; indicated by orange squares) for the ligation of two heme irons locating on each side of membranes, as predicted for other members of cytochrome b_{561} family. In our model, His67/His135 to be coordinated to heme b_H center, whereas His101/His174 to be coordinated to heme b_L center. The upper surface corresponds to the extracellular (or intravesicular or apical or luminal) side, whereas the lower surface corresponds to the cytosolic side.

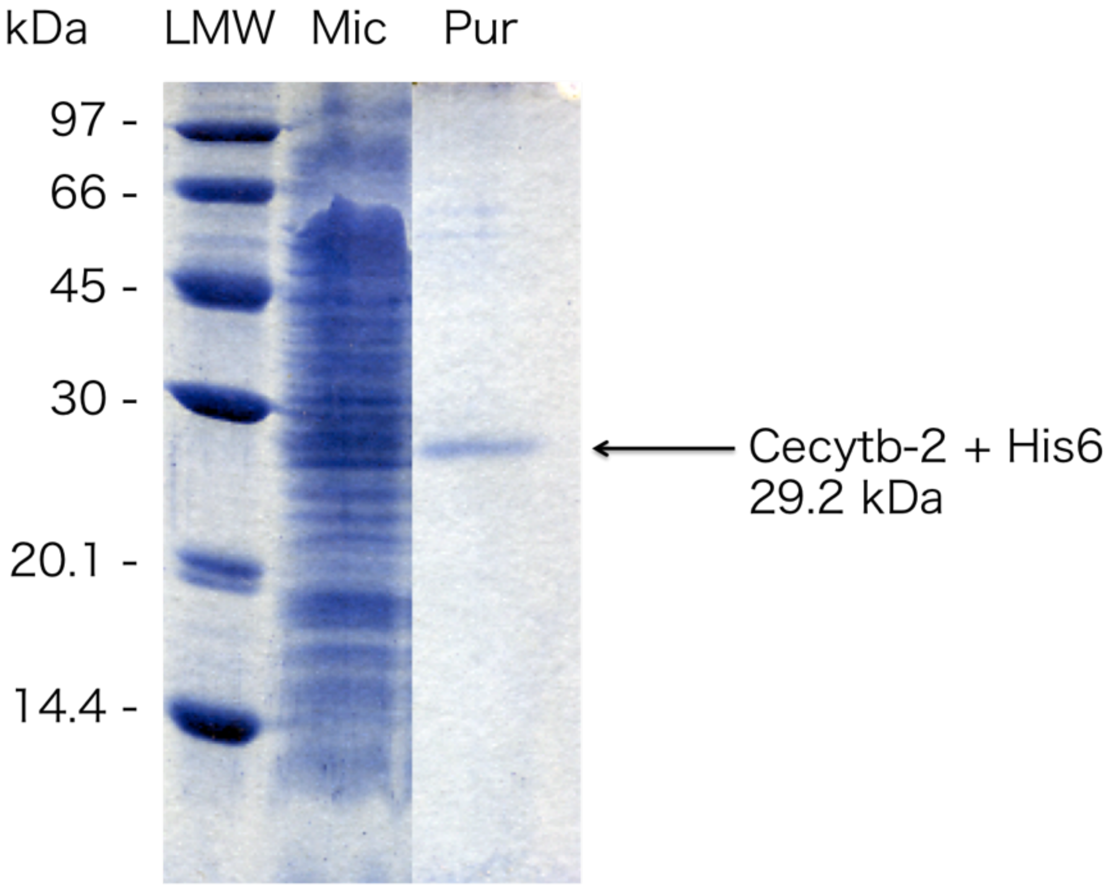

Figure S4. SDS-PAGE analysis of the purified Cecytb-2-H_6 recombinant protein. Purified recombinant Cecytb-2-H_6 protein (Pur; total, 1 μg) and the microsomal proteins (Mic; total, 110 μg) obtained from the recombinant *Pichia pastoris* cells were each analyzed on 15% polyacrylamide gel. The purified Cecytb-2 protein showed a single protein band at 29.2 kDa very close to the theoretical value (29.108 kDa) suggesting a highly purified state, although there were some impurities around 66 kDa. LMW indicates a molecular weight marker (LMW Marker Kit; GE HealthCare).

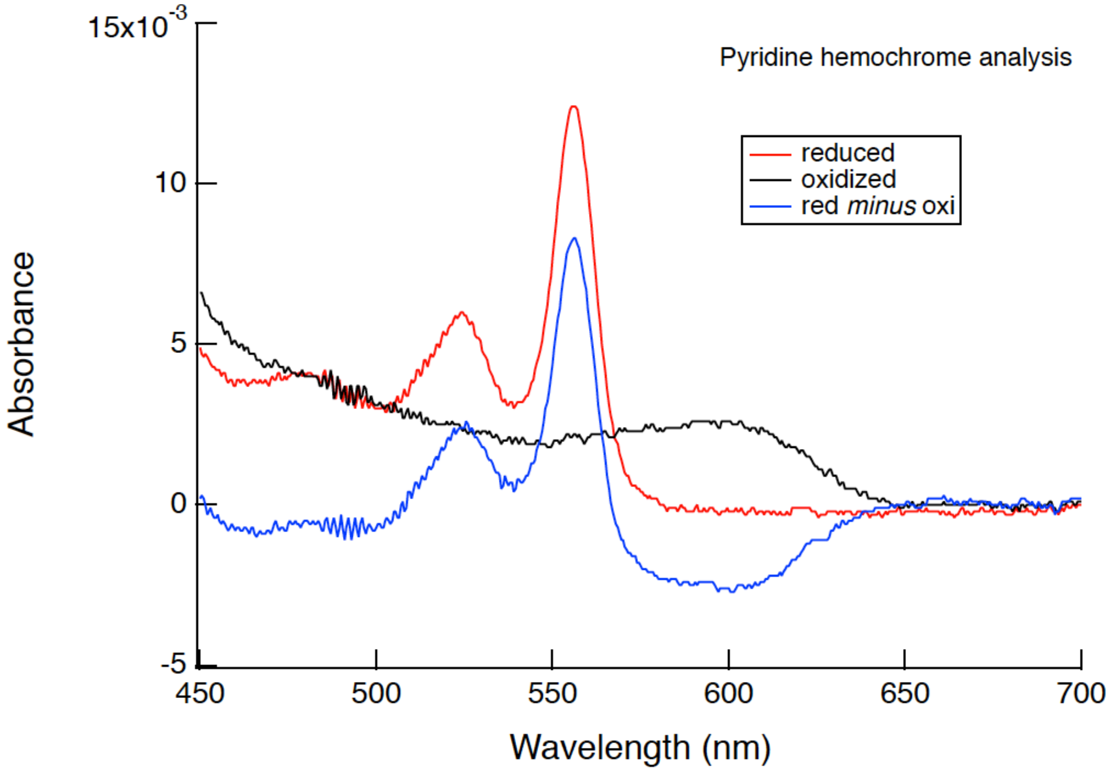

Figure S5. Pyridine hemochrome analysis of the purified Cecytb-2-H_6 protein. Pyridine hemochrome analyses were conducted on the purified Cecytb-2-H_6 protein in 50 mM K-Pi buffer (pH 7.4) containing 1% (w/v) OG. Heme *b* concentration was determined by using redox difference extinction coefficient value of $\varepsilon_{557-541}$=20.7 ($mM^{-1}cm^{-1}$). The spectra indicated above gave a value of 1.445 heme *b*/molecule (0.2145 mM of heme *b*/0.1484 mM of Cecytb-2 protein). Three independent analyses on the purified Cecytb-2-H_6 protein showed a mean value of 1.692 (± 0.177 SD) heme *b*/molecule.

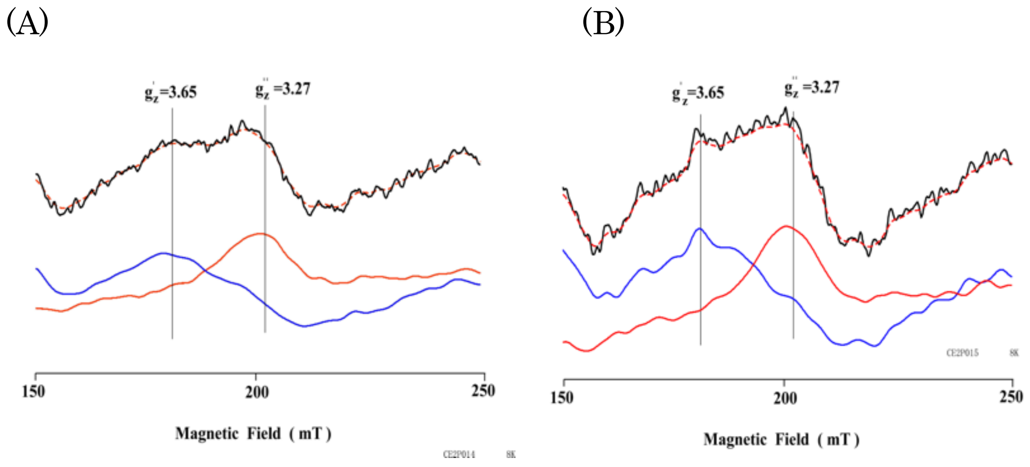

Figure S6. Spectral deconvolution of the g_z signals in EPR spectra measured at 8K for the purified Cecytb-2-H_6 protein in the air-oxidized state. Spectral deconvolution was conducted on the EPR spectra measured at 8K for two different preparations of purified Cecytb-2-H_6 protein ((A) CE2P014 and (B) CE2P015; one was the same with that shown in Figure 2) using GRAMS software (v. 8). In both analyses, the HALS-type g'_z=3.65 signal (blue line) and the usual rhombic g''_z=3.27 signal (red line) were identified in similar intensities.

(A) (B)

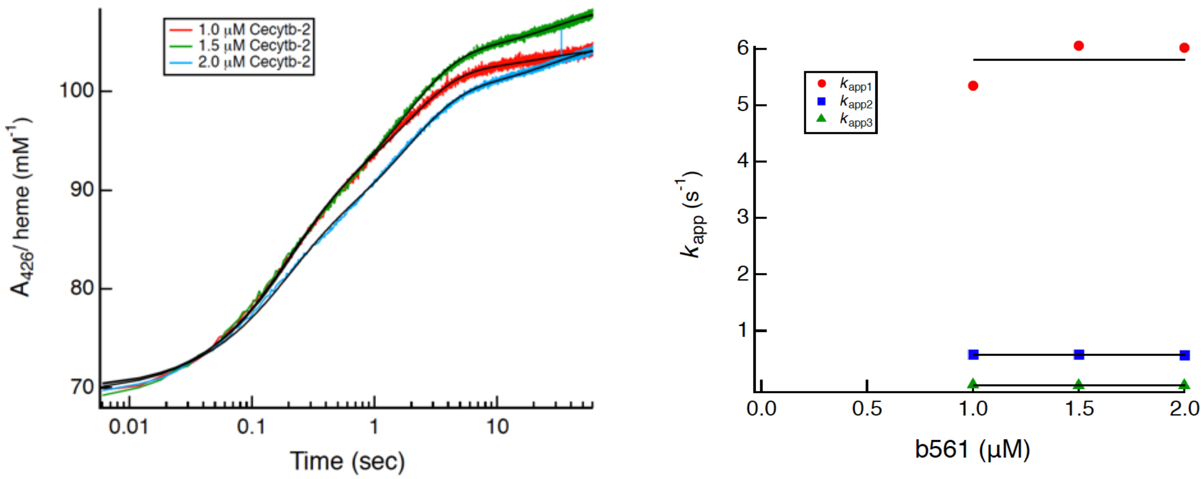

Figure S7. (A) Stopped-flow kinetic analyses on the electron transfer reaction of the purified Cecytb-2-H_6 protein with AsA at different protein concentrations. Air-oxidized purified form of Cecytb-2-H_6 protein in different concentrations (final, 2, 3, 4 μM in 50 mM potassium phosphate buffer, pH 7.4, containing 10 % (v/v) glycerol, 1 % (w/v) OG) were mixed with a fixed AsA concentration (2 mM) in a 1:1 volume ratio and following absorption changes at 426 nm were measured. Fitting to the data were conducted similarly to those described in Experimental Procedures section of the main text using a linear combination of three exponentials and the fitting curves are indicated in black for each data.

(B) Cecytb-2-H_6 protein-concentration dependency of three apparent rate constants (k_{app1}, k_{app2}, and k_{app3}) for the fitted curves as shown in panel (A).

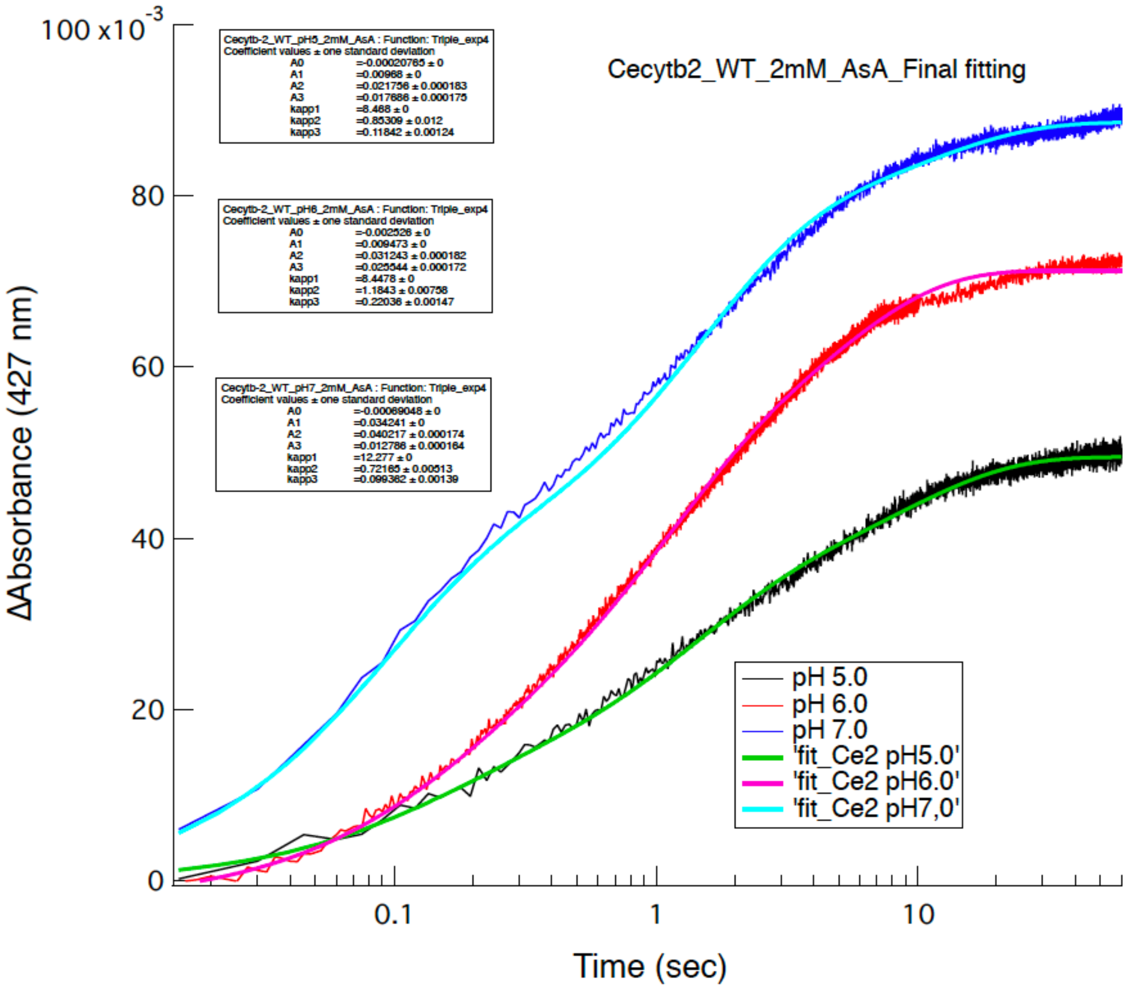

Figure S8. Stopped-flow kinetic analyses on the electron transfer reaction of the purified Cecytb-2-H_6 protein with AsA at different pH. Air-oxidized purified form of Cecytb-2-H_6 protein (2.0 μM) in different pH (50 mM potassium phosphate buffer for pH 6.0 and 7.0 and 50 mM sodium acetate buffer for pH 5.0, with each containing 1 % (w/v) OG) were mixed with a fixed AsA concentration (2 mM) in a 1:1 volume ratio and following absorption changes at 426 nm were measured. Fitting to the data were conducted similarly to those described in Experimental Procedures section of the main text using a linear combination of three exponentials and the fitted curves are indicated for each data. Fitted parameters are indicated in boxes in the panel.

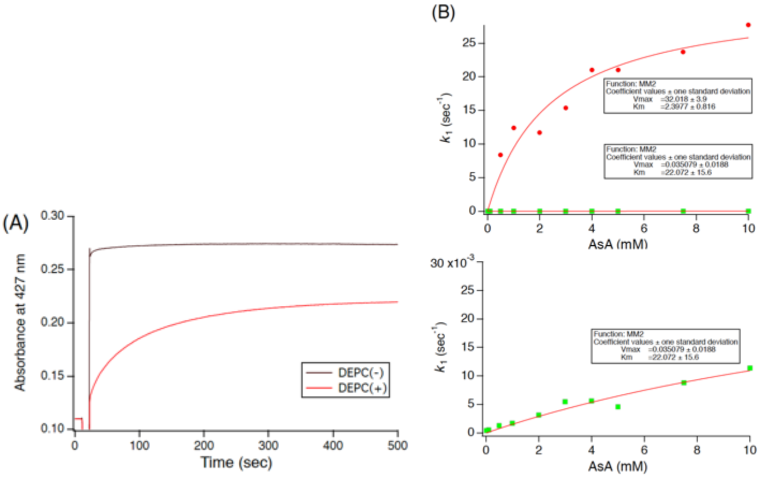

Figure S9. Effects of DEPC-treatment of Cecytb-2-H_6 on its reactivity with AsA.

(A) Effects of DEPC-treatment on Cecytb-2-H_6 for its reduction process with AsA and its final reduction level. Oxidized form of Cecytb-2-H_6 (1.4 μM) in 50 mM K-Pi buffer (pH 7.4) containing 0.1% DDM and 10% glycerol was treated with DEPC (final 0.5 mM by addition of 30 mM of EDTA in ethanol) at room temperature for 30 min, After the treatment, the sample was desalted by column chromatography to remove unreacted DEPC. Then, the DEPC-treated sample (or its control, treated with ethanol alone) was mixed with AsA (final 5 mM) and the absorbance change at 427 nm was recorded using a UV-vis spectrophotometer. For the control sample, reduction of the oxidized heme with AsA occurred rapidly within the mixing time. On the other hand, the DEPC-treated sample showed a significant inhibition in reduction with AsA for its initial rate and in the final reduction level (72.9% with the dithionite-reduced level as 100%). **(B) Effects of the DEPC-treatment of Cecytb-2-H_6 for its electron acceptance from AsA by a stopped-flow method and the Michaels-Menten type analyses on the apparent rate constants against the AsA concentration.** For the fitting of the stopped-flow data (not shown), a double exponential function ($A = A_0 + A_1 \exp(-k_1 t) + A_2 \exp(-k_2 t)$), instead of a triple exponential function, was used for the analysis to evaluate the very slow process of the electron transfer for the DEPC-treated Cecytb-2-H_6. The faster components in the equation were regarded as the electron transfer from AsA to the heme *b* center (heme b_L) on the cytosolic side and its apparent rate constant (k_1) were plotted against the AsA concentrations. Michaelis-Menten type analyses of the plot indicated that the V_{max} (for k_1) decreased by 900-folds (32 sec^{-1} *vs.* 0.035 sec^{-1}), whereas K_m increased by 9-folds (2.4 mM *vs.* 22 mM) (control, solid red circles; DEPC-treated, solid green squares; the lower panel shows an expansion of the upper panel).

(A)

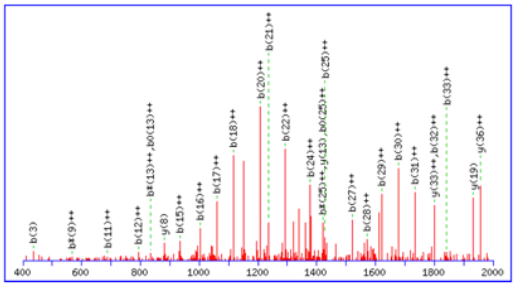

Label all possible matches ○ Label matches used for scoring ◉

Monoisotopic mass of neutral peptide Mr(calc): 4345.4235
Variable modifications:
K9 : Carbethoxy (CHKSTY)
H12 : Carbethoxy (CHKSTY)
Ions Score: 123 **Expect:** 4.9e-013
Matches : 29/436 fragment ions using 32 most intense peaks (help)

(B)

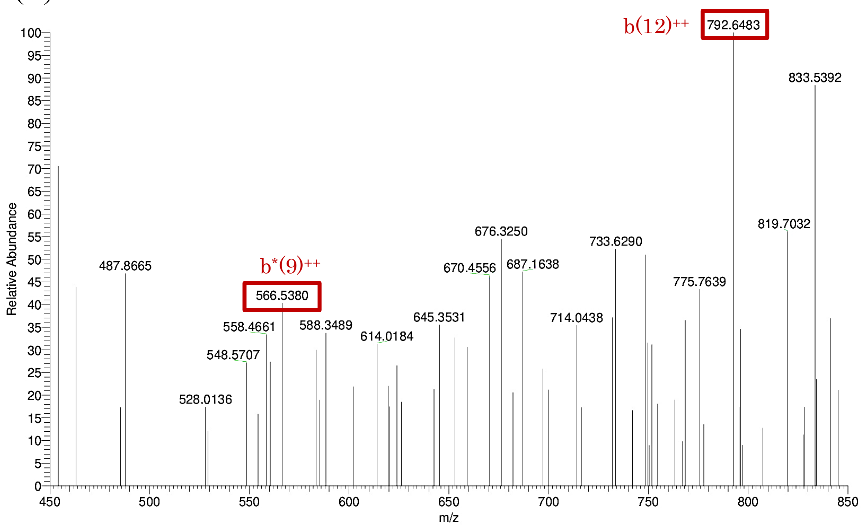

(C)

#	b	b++	b*	b* ++	b0	b0++	Seq	y	y++	y*	y* ++	y0	y0++	#
1	157.108	79.0578	140.082	70.5446			R							39
2	320.172	160.59	303.145	152.076			Y	4190.33	2095.67	4173.3	2087.16	4172.32	2086.66	38
3	435.199	218.103	418.172	209.59	417.188	209.098	D	4027.27	2014.14	4010.24	2005.62	4009.26	2005.13	37
4	506.236	253.622	489.209	245.108	488.225	244.616	A	3912.24	1956.62	3895.21	1948.11	3894.23	1947.62	36
5	634.331	317.669	617.304	309.156	616.32	308.664	K	3841.2	1921.1	3824.18	1912.59	3823.19	1912.1	35
6	747.415	374.211	730.388	365.698	729.404	365.206	I	3713.11	1857.06	3696.08	1848.54	3695.1	1848.05	34
7	860.499	430.753	843.472	422.24	842.488	421.748	I	3600.02	1800.52	3583	1792	3582.01	1791.51	33
8	947.531	474.269	930.504	465.756	929.52	465.264	S	3486.94	1743.97	3469.91	1735.46	3468.93	1734.97	32
9	1147.65	574.327	1130.62	565.814	1129.64	565.322	K	3399.91	1700.46	3382.88	1691.94	3381.9	1691.45	31
10	1260.73	630.869	1243.7	622.356	1242.72	621.864	L	3199.79	1600.4	3182.76	1591.89	3181.78	1591.39	30
11	1373.82	687.411	1356.79	678.898	1355.8	678.406	L	3086.71	1543.86	3069.68	1535.34	3068.7	1534.85	29
12	1582.9	791.951	1565.87	783.438	1564.88	782.946	H	2973.62	1487.32	2956.6	1478.8	2955.61	1478.31	28
13	1683.94	842.475	1666.92	833.962	1665.93	833.47	T	2764.54	1382.78	2747.52	1374.26	2746.53	1373.77	27
14	1754.98	877.994	1737.95	869.48	1736.97	868.988	A	2663.5	1332.25	2646.47	1323.74	2645.48	1323.25	26
15	1868.06	934.536	1851.04	926.022	1850.05	925.53	L	2592.46	1296.73	2575.43	1288.22	2574.45	1287.73	25
16	2005.12	1003.07	1988.1	994.552	1987.11	994.06	H	2479.37	1240.19	2462.35	1231.68	2461.36	1231.19	24
17	2118.21	1059.61	2101.18	1051.09	2100.2	1050.6	I	2342.32	1171.66	2325.29	1163.15	2324.3	1162.66	23
18	2231.29	1116.15	2214.26	1107.64	2213.28	1107.14	I	2229.23	1115.12	2212.2	1106.61	2211.22	1106.11	22
19	2302.33	1151.67	2285.3	1143.15	2284.32	1142.66	A	2116.15	1058.58	2099.12	1050.06	2098.14	1049.57	21
20	2415.41	1208.21	2398.39	1199.7	2397.4	1199.2	I	2045.11	1023.06	2028.08	1014.55	2027.1	1014.05	20
21	2472.43	1236.72	2455.41	1228.21	2454.42	1227.72	G	1932.03	966.517	1915	958.003	1914.02	957.511	19
22	2585.52	1293.26	2568.49	1284.75	2567.51	1284.26	L	1875	938.006	1857.98	929.493	1856.99	929.001	18
23	2642.54	1321.77	2625.51	1313.26	2624.53	1312.77	G	1761.92	881.464	1744.89	872.951	1743.91	872.459	17
24	2755.62	1378.32	2738.6	1369.8	2737.61	1369.31	I	1704.9	852.953	1687.87	844.44	1686.89	843.948	16
25	2856.67	1428.84	2839.64	1420.33	2838.66	1419.83	T	1591.81	796.411	1574.79	787.898	1573.8	787.406	15
26	2927.71	1464.36	2910.68	1455.84	2909.7	1455.35	A	1490.77	745.887	1473.74	737.374	1472.76	736.882	14
27	3040.79	1520.9	3023.77	1512.39	3022.78	1511.89	L	1419.73	710.369	1402.7	701.855	1401.72	701.363	13
28	3141.84	1571.42	3124.81	1562.91	3123.83	1562.42	T	1306.65	653.827	1289.62	645.313	1288.64	644.821	12
29	3240.91	1620.96	3223.88	1612.44	3222.9	1611.95	V	1205.6	603.303	1188.57	594.79	1187.59	594.298	11
30	3353.99	1677.5	3336.97	1668.99	3335.98	1668.49	I	1106.53	553.769	1089.5	545.255	1088.52	544.763	10
31	3467.08	1734.04	3450.05	1725.53	3449.07	1725.04	I	993.446	497.227	976.419	488.713	975.435	488.221	9
32	3598.12	1799.56	3581.09	1791.05	3580.11	1790.56	M	880.362	440.685	863.335	432.171	862.351	431.679	8
33	3685.15	1843.08	3668.12	1834.56	3667.14	1834.07	S	749.321	375.164	732.295	366.651	731.311	366.159	7
34	3786.2	1893.6	3769.17	1885.09	3768.19	1884.6	T	662.289	331.648	645.263	323.135	644.279	322.643	6
35	3900.24	1950.62	3883.21	1942.11	3882.23	1941.62	N	561.242	281.124	544.215	272.611			5
36	4014.28	2007.64	3997.26	1999.13	3996.27	1998.64	N	447.199	224.103	430.172	215.59			4
37	4085.32	2043.16	4068.29	2034.65	4067.31	2034.16	A	333.156	167.082					3
38	4142.34	2071.67	4125.31	2063.16	4124.33	2062.67	G	262.119	131.563					2
39							W	205.0972	103.0522					1

Figure S10. Identification of a doubly-*N*-carbethoxylated peptide derived from DEPC-treated Cecytb-2-H_6 by MS/MS MASCOT analysis.

(A) Identification of a doubly-*N*-carbethoxylated peptide upon digestion with α-chymotrypsin as RYDAKIIS<u>K</u>LL<u>H</u>TALHIIAIGLGITALTVIIMSTNNAGW by MS/MS MASCOT analysis. Red underlined residues in the sequence are corresponding to the well-conserved Lys98 and the fully-conserved His101. The latter residue is the axial ligand of the cytosolic heme b_L. In the spectrum, $b^*(9)^{++}$ peak indicates the *N*-carbethoxylation of Lys98 and the $b(12)^{++}$ peak indicates the *N*-carbethoxylation of His101, respectively.

(B) An enlarged view of the spectrum shown in (A) in 450~850 M/z region. The spectrum shows the $b^*(9)^{++}$ peak, indicating the *N*-carbethoxylation of Lys98, and the $b(12)^{++}$ peak, indicating the *N*-carbethoxylation of His101. Respective mass peaks are marked by red squares.

(C) Product mass ion list for the identification of the peptide RYDAKIIS<u>K</u>LL<u>H</u>TALHIIAIGLGITALTVIIMSTNNAGW by MS/MS MASCOT analysis as a doubly-*N*-carbethoxylated peptide. Numbers in red correspond to the mass peak identified in the MS/MS spectrum. The cells indicated with a background color in orange correspond to the $b^*(9)^{++}$ peak and $b(12)^{++}$ peak, respectively.

	DEPC HPLC	Area (%)	C	C	C	V	V	V	V	V	V	V	V	V	V	C	C	C
CGb561 (bovine)			–	–	–	Ser21	Gln22	Ala30	Ala34	Ser44	Ala45	–	–	–	His54	Phe64	Tyr73	Phe76
CGb561 (human)			–	–	–	Ser20	Gln21	Ala29	Ala33	Ser43	Asp44	–	–	–	His53	Phe63	Tyr72	Phe75
Zmb561 (Z.mays)			–	–	–	Ala16	His17	Ala25	Val29	Ser42	Thr43	Lys45	–	–	His52	Ile62	Tyr71	Leu74
CYB561B(A.thal)			–	–	–	Val15	Arg16	Ala24	Thr28	Ser41	Asp42	–	–	Asp45	His51	Leu61	Tyr70	–
Dcytb (human)			–	–	–	Ala16	Leu17	Ile25	Val29	Ser46	Ala47	–	–	–	His50	Phe60	Tyr69	Thr77
**Cecytb-2**			**Ser2 N-**	**Ser2**	**Ser3**	**Thr23**	**His24**	**Cys32**	**Tyr36**	**Ser49**	**Lys50**	**Lys52**	**Lys57**	**Lys61**	**His67**	**Tyr77**	**Tyr86**	**Tyr89**
Trypsein＋V8	DEPC＋90min	AA(none)	0	0	0	70151	70151	70151	70151	556667	556667	0	997956	185253	25286210	25170775	20085717	43520
		AA(Carbethoxy)	0	0	0	0	0	0	0	0	0	1341751	433951	24021	307683	423118	2049847	152812
		total AA	0	0	0	70151	70151	70151	70151	556667	556667	1341751	1431907	209274	25593893	25593893	22135564	196332
		Carbethoxy(%)				0	0	0	0	0	0	100	30.3	11.5	1.2	1.7	9.3	77.8
α-chymotrypsin	DEPC＋90min	AA(none)	0	79571	0	10141779	2378898	1902305	1360465	9572886	1574664	427267	548874	2777460	6468333	7571433	0	0
		AA(Carbethoxy)	79571	0	79571	52917	7815798	76050	52918	1574664	9572886	10720283	10598676	17891036	1945814	169266	0	0
		total AA	79571	79571	79571	10194696	10194696	1978355	1413383	1.1E+07	11147550	11147550	11147550	20668496	8414147	7740699	0	0
		Carbethoxy(%)	100	0	100	0.5	76.7	3.8	3.7	14.1	85.9	96.2	95.1	86.6	23.1	2.2		

	DEPC HPLC	Area (%)	C	C	C	C	C	C	C	V	C	V	V	V	C	C	C	C
CGb561 (bovine)			Asn78	Lys81	Thr84	Lys85	His88	Gly89	Hs92	His122	His161	–	Val200	Leu205	Ala213	Leu229	Gln230	–
CGb561 (human)			Asn77	Lys80	Thr83	Lys84	His87	Gly88	His91	His121	His160	Thr193	Val199	Leu204	Gly212	Ser228	Gln229	–
Zmb561 (Z.mays)			Thr76	His79	Thr82	Lys83	His86	Leu87	His90	Hid120	His159	Gly189	Phe198	Thr203	Gly211	Glu225	Glu226	–
CYB561B(A.thal)			–	Lys77	Lys80	Lys81	His84	Leu85	Gln88	His118	His157	–	Met197	Met202	Gly210	Ser222	Gly223	–
Dcytb (human)			Lys79	Lys82	Met85	Lys86	His89	Ala90	Asn93	His120	His159	Ala193	Val199	Leu204	Gly212	Lys228	Glu229	–
**Cecytb-2**			**Tyr91**	**Lys94**	**Ser97**	**Lys98**	**His101**	**Thr102**	**His105**	**His135**	**His174**	**Cys204**	**Tyr216**	**Ser221**	**Thr229**	**Lys245**	**Thr246**	**Lys251**
Trypsein＋V8	DEPC＋90min	AA(none)	0	0	0	0	0	0	0	0	66269	0	4964090	6140090	7737229	1421855	6948944	4310888
		AA(Carbethoxy)	0	0	0	0	0	0	0	0	0	0	2773139	1597139	0	5152038	0	1900296
		total AA	0	0	0	0	0	0	0	0	66269	0	7737229	7737229	7737229	6573893	6948944	6211184
		Carbethoxy(%)									0		35.8	20.6	0	78.4	0	30.6
α-chymotrypsin	DEPC＋90min	AA(none)	1562645	1292114	1630905	578437	660585	1024688	1196918	287368	0	4204189	0	2824386	2824386	1535762	1535762	0
		AA(Carbethoxy)	257083	1177660	838869	1891337	1106296	742193	588780	0	0	9465808	0	0	0	1535762	1535762	0
		total AA	1819728	2469774	2469774	2469774	1766881	1766881	1785698	287368	0	13669997	0	2824386	2824386	3071524	3071524	0
		Carbethoxy(%)	14.1	47.7	34.0	76.6	62.6	42.0	33.0	0		69.2		0	0	50.0	50.0	

C facing cytosolic side
V facing intravesicular side

heme *b* ligand (fully-conserved) residues
highly-conserved residues

Extent of carbethoxylation(%)
>>40%
20～40%
<20%

Figure S11. Summary of carbethoxylated residues of Cecytb-2-H_6 upon the DEPC-treatment. Identification and quantification were conducted by MS/MS MASCOT analysis. In the most upper line, cytosolic side (C) and intravesicular (= apical or luminal) side (V) were indicated; in the following six lines, conservation of the amino acid residues of five representative members of the cytochrome b_{561} protein family corresponding to those of Cecytb-2 with carbethoxylated upon the DEPC-treatment were indicated. In addition, well-conserved residues among the cytochrome b_{561} family members were marked with a background color in light blue, whereas four fully-conserved His residues responsible for the binding to two heme *b* centers are marked with a background color in light red. The extent of carbethoxylation for each amino acid residue (in %) were calculated based on the MS/MS MASCOT analyses for the entire eluates from online-HPLC column and were marked with a background color of orange or yellow.

(A)

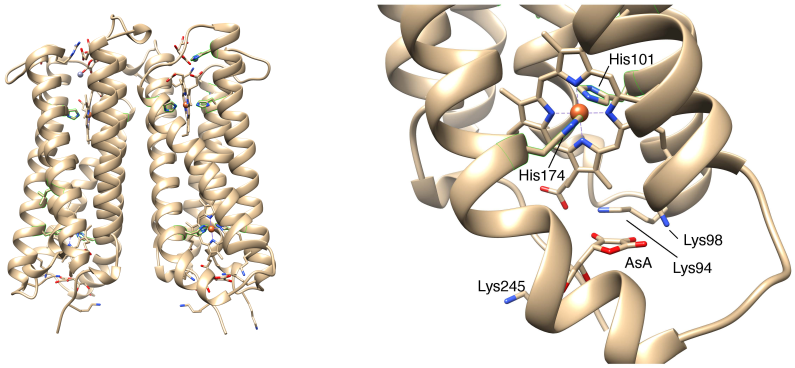

(B)

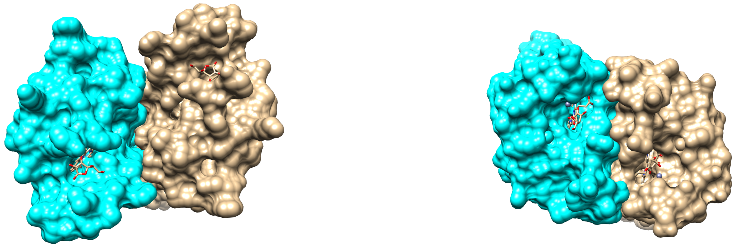

Figure S12. The putative structural models of Cecytb-2 protein.

(A) Side views of calculated structure of Cecytb-2 in comparison with crystal structure of human Dcytb. (Left) In an original dimeric crystal structure of Dcytb protein (5ZLG.pdb) (Ganasen *et al.*, 2018)[24], one molecule (left part) was replaced with a calculated structure of Cecytb-2 with the cytosolic heme b_L in a lower position. (Right) A closer view of the calculated structure of Cecytb-2 protein in cytosolic side, showing an AsA molecule with two Lys residues (Lys 94 and Lys98) nearby and the heme b_L center being coordinated with fully-conserved His101and His 174.

(B) Views from cytoplasmic surface (left) and from apical surface (right) of Cecytb-2 in comparison with human Dcytb. (Left) A view from cytoplasmic surface. Calculated structure of Cecytb-2 (left, light blue) was compared with the crystal structure of Dcytb (right, yellow). An AsA molecule and a part of heme b_L center can be seen in the crevice of each molecule. (Right) A view from apical (or luminal) surface. Calculated structure of Cecytb-2 (left, light blue) was compared with the crystal structure of Dcytb (right, yellow) (5ZLG.pdb) (Ganasen *et al.*, 2018)[24]. An AsA molecule, a part of heme b_H center, and a Zn^{2+} ion (blue) can be seen in the crevice of each molecule.

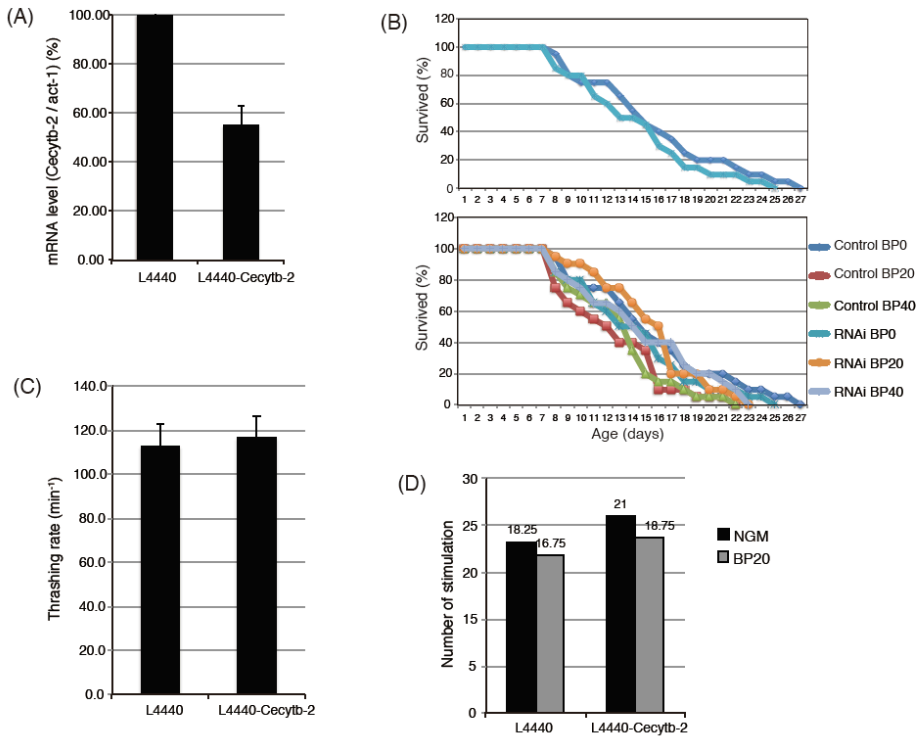

Figure S13. Effect of RNAi on the Cecytb-2 gene expression and the resulting phenotypes of *C. elegans*. Knock-down of the *Cecytb-2* gene by feeding RNAi method (A) did not show any appreciable changes in their phenotypes, such as their life span (B), motility (C), and memory (D). **(A)** The expressed *Cecytb-2* mRNA level was analyzed by PCR and was compared with that of actin mRNA level transcribed from *act-1* gene, indicating 50 - 45% decrease upon knock-down by feeding RNAi method (N=20). **(B)** Life spans of RNAi-treated and control N2 worms were compared by culturing on NGM plates without BP or with 20 mM or 40 mM of BP. The survival curves indicated that the RNAi on the *Cecytb-2* gene expression did not affect their life spans. **(C)** RNAi-treated and control N2 worms were compared in their thrashing rate activities (min^{-1}) in M9 buffer. The worms were each cultured on NGM plates without BP or in the presence of 20 mM of BP. The RNAi-treatment did not cause any appreciable difference in their motility. **(D)** RNAi-treated and control N2 worms were each cultured on NGM plates without BP. Then, the maximum number of physical stimulations required to reach the dormant stage was compared. The test showed that there was no difference between the RNAi-treated worms and the control N2 worms.

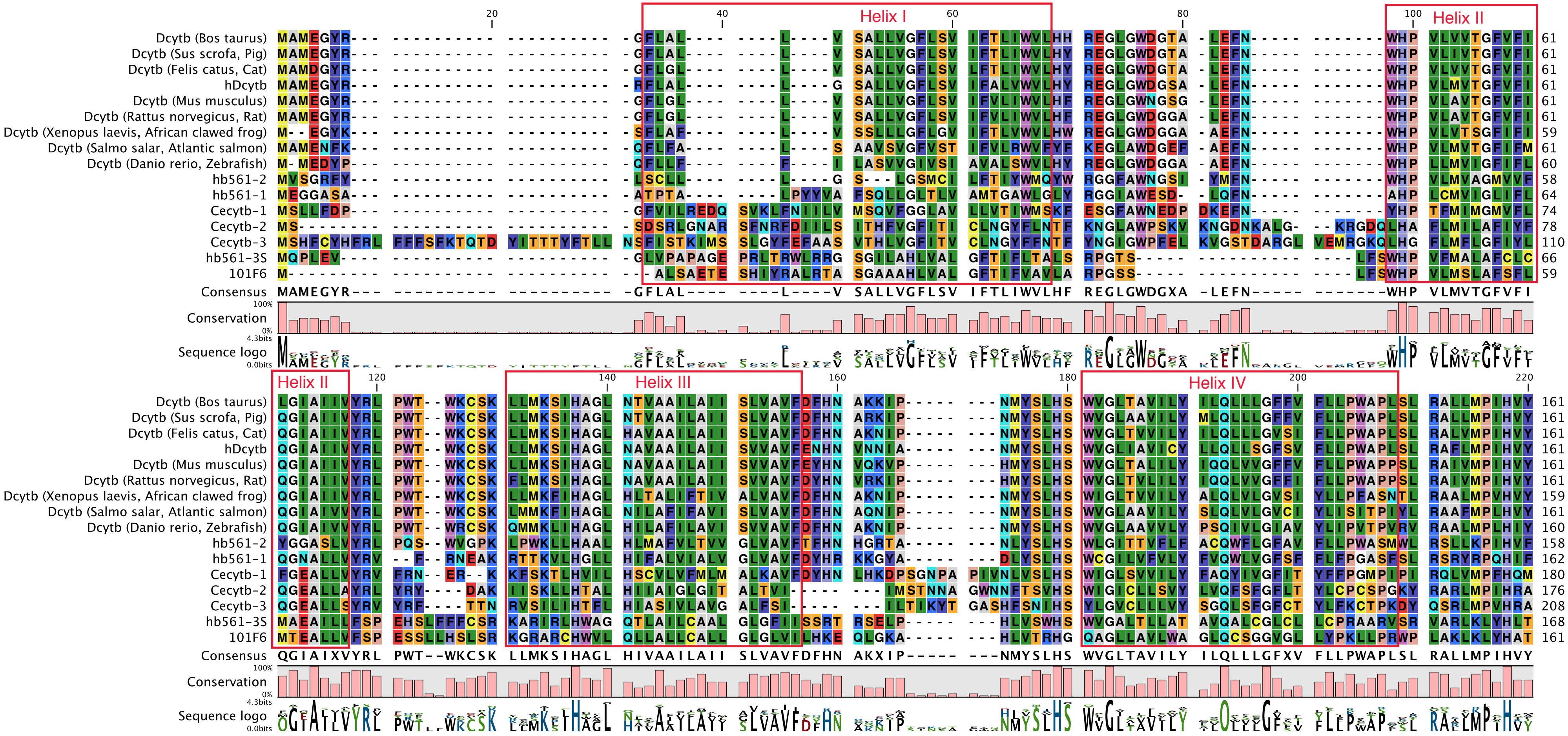
Helix I
Helix II
Helix III
Helix IV
Dcytb (Bos taurus)
Dcytb (Sus scrofa, Pig)
Dcytb (Felis catus, Cat)
hDcytb
Dcytb (Mus musculus)
Dcytb (Rattus norvegicus, Rat)
Dcytb (Xenopus laevis, African clawed frog)
Dcytb (Salmo salar, Atlantic salmon)
Dcytb (Danio rerio, Zebrafish)
hb561-2
hb561-1
Cecytb-1
Cecytb-2
Cecytb-3
hb561-3S
101F6
Consensus
Conservation
Sequence logo

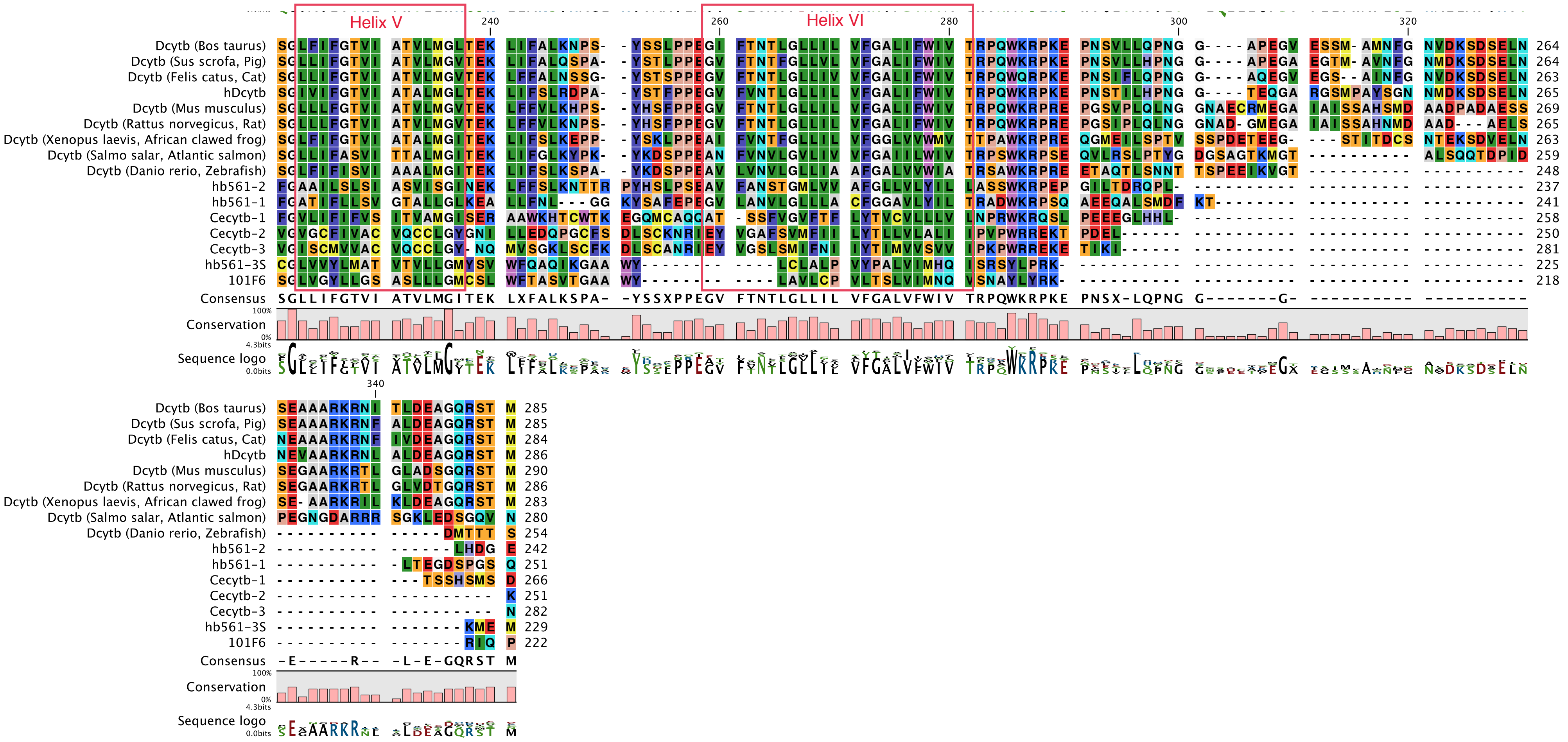
Helix V
Helix VI
Dcytb (Bos taurus)
Dcytb (Sus scrofa, Pig)
Dcytb (Felis catus, Cat)
hDcytb
Dcytb (Mus musculus)
Dcytb (Rattus norvegicus, Rat)
Dcytb (Xenopus laevis, African clawed frog)
Dcytb (Salmo salar, Atlantic salmon)
Dcytb (Danio rerio, Zebrafish)
hb561-2
hb561-1
Cecytb-1
Cecytb-2
Cecytb-3
hb561-3S
101F6
Consensus
Conservation
Sequence logo

Figure S14. Multiple alignments of amino acid sequences of human cytochromes b_{561} (CGcytb, Dcytb, and Lcytb) and their close homologs of *C. elegans* (Cecytb-1, Cecytb-2, and Cecytb-3). Multiple alignments of hDcytb (and its homologs in other species) and other human cytochromes b_{561} (CGcytb = hb561-1, Lcytb = hb561-2, 101F6, and hb561-3S) and their homologs in *C. elegans* (Cecytb-1, Cecytb-2, Cecytb-3) were conducted using CLC-Main Workbench software (v. 6.8) (CLC Bio). Particular interest is the distribution of negatively-charged residues (D and E, indicated in a red background) for hDcytb and its homologs, which may assist in directing ferric ions toward their active sites for the ferric reductase reaction. However, these negatively-charged residues and the proposed interacting residues with Zn^{2+}-ascorbate (AsA) (as a ferric ion model) in hDcytb (Asn107, His108, Tyr117, and Phe184) based on X-ray structure (Ganasen et al., 2018)[24] are not well conserved in Cecytb-2, except for Tyr117, which is a part of a putative MDA radical binding sequence (Tsubaki *et al.*, 2005)[5].

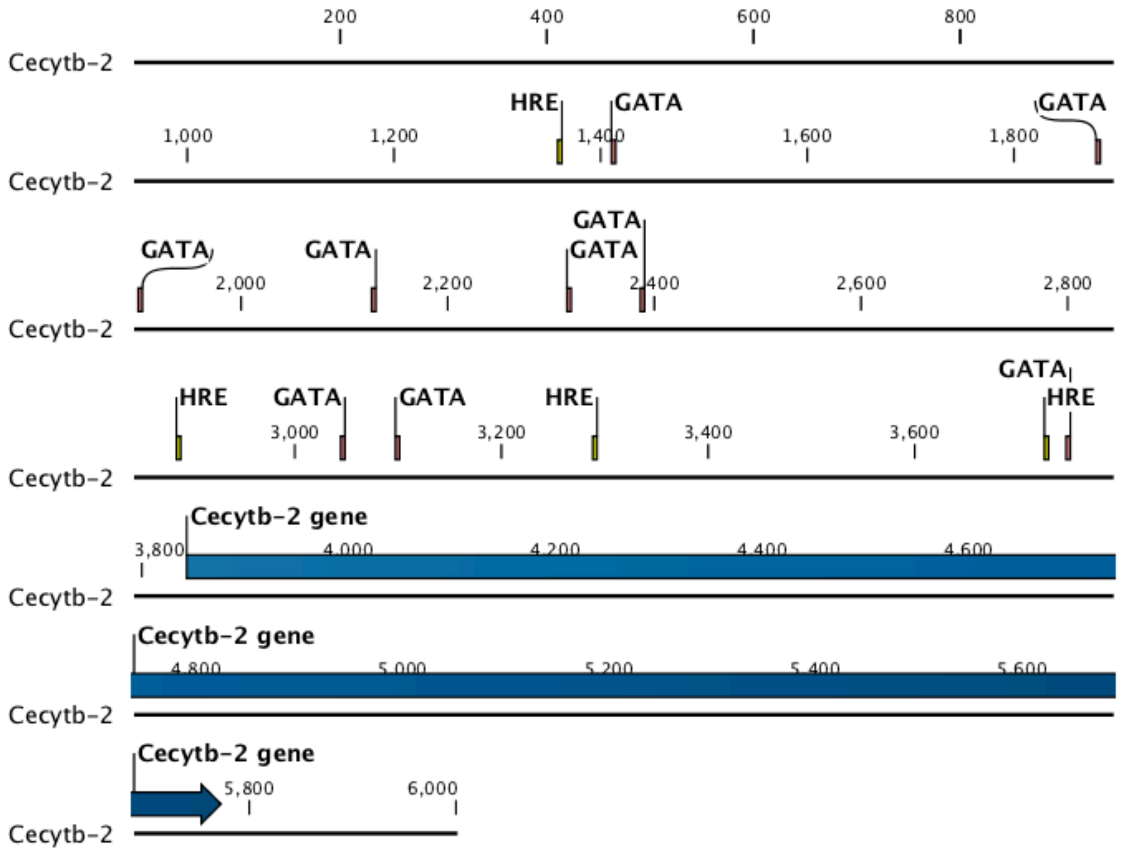

Figure S15. Genome map of *Cecytb-2* gene and its 5' upstream region up to 3,000 bp. The 5' upstream region of *Cecytb-2* gene (*F39G3.5*) was analyzed assuming that the region 3,000-bp upstream of the start codon of *Cecytb-2* gene contains its promoter. We found that there were four HRE sequences and nine GATA sequences in this region, suggesting that the coupling of Cecytb-2 protein to the iron uptake system might be also regulated in the gene expression level.
